# Developmental differences in processing the valence and magnitude of incentive cues: Mid-adolescents are more sensitive to potential gains than early- or late-adolescents

**DOI:** 10.3758/s13415-021-00978-7

**Published:** 2022-01-18

**Authors:** Nicola K. Ferdinand, Efsevia Kapsali, Marc Woirgardt, Jutta Kray

**Affiliations:** 1 Department of Psychology, Bergische Universität Wuppertal, Wuppertal, Germany; 2 Department of Psychology, Saarland University, Saarbrücken, Germany

**Keywords:** Task-switching, Event-related potentials, Childhood, Adolescence, Incentives

## Abstract

Recent research has focused on the interaction between motivation and cognitive control and shown that both are important for goal-directed behavior. There also is evidence for developmental differences in the sensitivity and behavioral effectiveness of incentives, showing that mid-adolescents might be especially susceptible to rewards. Further pursuing this line of research, the present study examined developmental differences in incentive processing and whether these potential differences also would correspond to changes in cognitive control. We compared the processing of high and low potential gains and losses in early-, mid-, and late adolescents by means of event-related potentials (ERPs) and examined whether these incentives also led to specific performance differences in task-switching. We expected that potential gains compared to potential losses and high compared to low incentives would lead to more preparatory updating as reflected in the P3b and consequently to better task performance and smaller global and local switch costs as indicators of cognitive control in all age groups. Furthermore, we expected that mid-adolescents should be especially sensitive to high gains and thus show the most pronounced enhancements in task performance and global and local switch costs in trials with high gains, respectively. Our results corroborate the idea of a special sensitivity to high rewards during mid-adolescence. The analysis of ERPs showed age-related differences in the processing of incentive cues that also varied with cognitive control demands. However, the different incentives did not impact age-related differences in indices of cognitive control, but had a general effect on response speed.

## Introduction

In recent years, researchers have begun to systematically investigate interactions between cognitive control and motivational functioning both at the behavioral level as well as at the neuronal level (for a review, Pessoa, 2017; Yee & Braver, 2018). While certainly both, motivation and cognitive control, are essential for goal-directed behavior, there is no consensus so far on how to exactly define and distinguish their relative impact on behavior. Generally, most researchers would agree on the view that external incentives (e.g., monetary gains, social approval, or positive feedback) will lead to changes in motivational states that in turn induce changes in cognitive control processes which can then be observed in changes in behavior (Yee & Braver, 2018). Additionally, there is evidence for developmental differences in the sensitivity to and the behavioral effectiveness of incentives, and it is a recurring theme in the recent literature that mid-adolescents might be especially susceptible to potential rewards (for reviews, see Kray et al., 2018; Richards et al., 2013). For this reason, we investigated how the valence (positive or negative) and the magnitude (high or low) of incentives are processed in different age groups (early-, mid-, and late-adolescents) and whether these potential differences would also be evident in terms of specific changes in task performance related to core components of cognitive control, i.e., the ability to flexibly switch between simple cognitive tasks. Hence, we examined whether developmental differences in incentive processing also would manifest in respective behavioral differences.

### Sensitivity to incentives in mid-adolescence

Investigating the interplay between motivation and cognition is particularly important during adolescence. This developmental period has been linked to a maturational imbalance between the two brain systems underlying motivational processing and cognitive control, resulting in an increased susceptibility to motivational conditions (Casey et al., 2008; Steinberg, 2008; Kray, Ritter & Müller, 2020; Luna & Wright, 2016; for a review, see Shulman et al., 2016). According to the so-called dual systems models (Shulman et al., 2016), the relative dominance of “hot” affective-motivational influences over “cold” aspects of cognitive functioning, particularly during mid-adolescence, may explain increases in sensation seeking and risk taking typically observed during this period (Poon, 2018).

In line with these models, a number of recent studies found evidence for developmental changes in the brain’s sensitivity to potential rewards (for reviews, see Kray et al., 2018; Richards et al., 2013). For instance, Van Leijenhorst et al. (2010) examined the anticipation and the delivery of monetary gains in a gambling task and found larger striatal activity to the delivery of rewards in 14–15-year-old adolescents compared with younger children and young adults. Similarly, Cohen et al. (2010) found a hypersensitive response to unpredicted rewards in the striatum and angular gyrus of adolescents aged 14–19 years compared with younger children and adults in a probabilistic learning task. Galvan et al. (2006) also found larger activation in reward-related brain regions in 13–17-year-old adolescents relative to younger children and young adults.

In contrast, the results concerning the processing of losses or penalties are rather mixed. Galvan and McGlennen (2013) studied brain activations during the anticipation and the delivery of primary incentives in a passive reward-delivery paradigm and found stronger activations in the ventral striatum of 13–17-year-old adolescents in response to sugary (i.e., rewards) and salty (i.e., penalties/losses) liquids compared with water. However, May et al. (2004) examined the delivery of monetary gains and losses in a card-guessing game with children and adolescents aged 8–18 years. Contrary to the results by Galvan and McGlennen (2013), this study found larger and later peak activations in the striatum and orbitofrontal cortex to gains than losses and no reliable age differences in these activations (for a similar result, see Van Duijvenvoorde et al., 2014).

In addition to the valence, the magnitude of rewards has been examined. For example, Bjork et al. (2004) applied a monetary incentive delay task in which cues indicated potential gains and losses that varied in magnitude (low, middle, high). The results revealed that in particular the ventral striatum was sensitive to the magnitude of anticipated gains and less sensitive to anticipated losses. Moreover, adolescents recruited the right ventral striatum and amygdala less than young adults during the anticipation of gain incentives. In contrast, no age differences in brain activation changes of anticipated losses were found. In a second study, however, Bjork et al. (2010) found better performance with increasing magnitude of incentives but no interaction with its valence (gains or losses) or with age. In line with their previous findings, the authors reported reduced brain activation (here in the nucleus accumbens) in adolescents compared with adults during the anticipation of monetary gains and losses (see also, Geier et al., 2010).

In summary, although the results on the valence and the magnitude of incentives are rather mixed, most studies found evidence for a special sensitivity to motivational influences in mid-adolescence. These influences are crucial to investigate, not only because they could explain risk taking and sensation seeking during adolescence, but also because they could be used in a beneficial way to affect the effective implementation of cognitive control processes.

### Development of cognitive control

The term cognitive control encompasses a wide range of higher order cognitive functions guiding lower level sensory and motor processes according to internal goals and environmental conditions (Friedman & Miyake, 2017; Miller & Cohen, 2001; Miyake et al., 2000). Cognitive control processes have been extensively studied using the task-switching paradigm (for reviews, see Gajewski et al., 2018; Grange & Houghton, 2014; Jamadar et al., 2015, Kiesel et al., 2010; Kray & Ferdinand, 2013). In this paradigm, participants are instructed to perform two (or more) different simple cognitive tasks, such as categorizing pictures or naming colors. In single-task blocks, they only perform one of these tasks, whereas in mixed-task blocks, they are instructed to switch between these tasks either in a predictable order or by means of a task-cue at the beginning of a trial.

In this paradigm, two types of costs reflecting different aspects of cognitive control can be derived. Global switch costs (also termed mixing or general switch costs) are defined as the difference in performance between mixed- and single-task blocks. These costs are considered to reflect the ability to deal with being in a switching situation (Kray & Dörrenbächer, 2019), i.e., selecting between task-sets and maintaining the one that is currently needed, compared with maintaining only one relevant task-set (see also global alternation cost in Meiran, 2000). Thus, global switch costs appear to depend more on the ability to maintain and manipulate multiple task-sets in working memory rather than reflect the switching process per se (Kray & Lindenberger, 2000). Local switch costs (also referred to as specific switch costs) are defined as the difference in performance between switch and repeat trials within mixed-task blocks. They are assumed to reflect the ability to disengage from a previous task rule and to shift to another one and are therefore more closely linked to the switching process per se.

Task switching costs as well as age differences therein are well documented in the literature (Koch et al., 2018; Kiesel et al., 2010; Grange & Houghton, 2014). Specifically, developmental studies report larger global switch costs in children and adolescents compared with young adults (Crone et al., 2006; Karbach & Kray, 2007; Kray et al., 2004; Manzi et al., 2011; Reimers & Maylor, 2005). Research findings regarding age differences in local switch costs are less consistent. Some studies report no reliable age differences (Crone et al., 2006; Karbach & Kray, 2007; Kray et al., 2004; Manzi et al., 2011; Reimers & Maylor, 2005), whereas others found declining local switch costs with increasing age (Cepeda et al., 2001; Davidson et al., 2006; Gupta et al., 2009; Huizinga et al., 2006). These findings suggest that the ability to maintain and select between different task-sets improves over childhood, while the ability to actually switch between the different tasks is more age-invariant (see McKewen et al., 2019 for a recent finding in the opposite direction).

In a similar vein, an event-related potential (ERP) study using a cued task-switching paradigm found that 9–10-year-old children showed a delayed anticipatory task-set updating, compared with 13–14-year-old adolescents and young adults, as indexed by a delayed P3b after the task cue (Manzi et al., 2011). The authors proposed that this could be the reason for the larger global switch costs in reaction time in this age group. In contrast, local switch costs appeared to be age-invariant. However, ERPs indicated that children did not only update task representations in switch trials, where it was necessary, but also on repetition trials, which might explain why no age-related differences in local switch costs were found and suggests that the process of reconfiguring the task-set is not yet mature. In a similar vein, Karayanidis et al. (2013) also found that children and early adolescents processed repeat trials more like switch trials, especially under conditions of high stimulus interference, resulting in little differentiation between switch and repeat trials within mixed blocks and, thus, reduced local switch costs. Overall, there is evidence for larger task-switching costs in childhood and early adolescence compared with adultlike performance, with the magnitude of such developmental differences varying as a function of the task parameters (Karayanidis et al., 2013), as well as the respective operationalization applied (Cragg & Chevalier, 2012).

### Impact of incentives on cognitive control and performance

Although studies examining age differences in the effects of incentives on task-switching performance are fully missing, a number of studies have examined young adults. For example, Umemoto and Holroyd (2015, b) employed an incentivized task-switching design where only one of two simple tasks was associated with the prospect of reward. The authors found that both accuracy and response latency substantially improved in the rewarded task relative to the nonrewarded task. These reward-related improvements were largest on switch compared with repeat trials, resulting in reduced switching costs in the rewarded condition. This pronounced effect of rewarding incentives has been interpreted as reflecting a specific facilitation of the task-switching ability (Kleinsorge & Rinkenauer, 2012). Similarly, Shen and Chun (2011) found that participants responded faster on high relative to low reward trials during task-switching and that this speed-up was most pronounced when participants were required to switch between the tasks rather than repeat the same task. However, this reward-induced modulation of the task-switching ability was only evident when the reward prospect increased relative to the previous trial. That is, although constantly high rewards were most effective in speeding up overall reaction times and maintaining response accuracy, only the relative increase of reward prospect led to a decrease of switching costs (Shen & Chun, 2011; for a similar finding on the modulation of the flexibility-stability balance depending on the immediate reward history in a voluntary task-switching paradigm, see Fröber, Pfister, & Dreisbach, 2019). In line with this, Otto and Vassena (2020) found similar reductions in switching costs associated with high rewards. They additionally demonstrated that these reward-induced switching cost reductions are sensitive to the reward context, such that the same (absolute) reward amount may lead to higher cognitive flexibility in reward-poor versus reward-rich motivational contexts, consistent with the notion of a context-dependent value of incentives.

Capa et al. (2013) combined an incentivized, cued task-switching paradigm with an ERP approach. In their behavioral data, they found evidence for general enhancements in terms of higher response accuracy and shorter response latencies in conditions where performance contingent rewards were anticipated. However, unlike the findings reported above, no specific interaction with trial type, i.e., no reward-related modulation of switching costs, was obtained. On the neuronal level, prospective rewards were linked to a larger frontocentral contingent negative variation (CNV) after the presentation of the task cue, suggesting incentive-induced enhancements in task-preparation processes. Moreover, larger probe-locked P3b amplitudes in trials with prospective rewards indicated a larger allocation of working memory resources during response selection and execution, which was significantly correlated with the reaction time benefits on the behavioral level. However, again, no specific modulations of switching costs were found.

Applying representational similarity analysis (RSA) to electroencephalogram (EEG) data, Hall-McMaster et al. (2019) examined whether incentives can shape the neural coding of task rules during task-switching. They found that participants gave faster and more accurate responses on trials with high incentives. However, on the behavioral level, no significant modulation of task-switching costs by reward prospect was obtained. On the neuronal level, potential rewards led to a more reliable coding of the active task-rule, which is consistent with the view that rewarding incentives enhance task preparation processes. Critically, this effect was more pronounced on switch trials, where participants needed to flexibly shift between the two task-rules. Hence, incentives appeared not only to promote task encoding, in general, but also support cognitive control, presumably by optimizing the neural separation and reducing interference between competing task-rules.

Using a different paradigm to examine cognitive control processing in young adults, Schmitt et al. (2015) used an incentivized AX-CPT combining both a gain and a loss condition. They reported a flexible modulation in the predominant manner of context updating as a function of incentive valence in young adults: incentives signaling the prospect of reward appeared to speed-up responding. This effect was corroborated by an enhanced updating of the context cue information on the neuronal level and was attributed to an increased employment of preparatory control in the face of potential gains. However, on the neuronal level, young adults also exhibited an increased employment of cognitive control in the form of increased conflict resolution and performance monitoring after the presentation of the target stimuli, whenever incorrect responding would lead to the loss of a bonus. This bias toward a more cautious responding to avoid negative outcomes may have in turn contributed to the valence-related modulation of response latency, resulting in a relative slowdown of responding (Schmitt et al., 2015; Schmitt et al., 2017).

To summarize, although no developmental studies have examined the influence of incentives on task-switching performance, studies with adult samples found evidence that incentives can not only lead to general performance benefits in terms of faster responding but also to smaller switching costs, in line with the view that rewards may foster top-down control (Kleinsorge & Rinkenauer, 2012; Otto & Vassena, 2020; Shen & Chun, 2011; Umemoto and Holroyd, 2015, b). Moreover, studies using electrophysiological approaches have shown that these beneficial effects of incentives on cognitive control seem to be at least partly due to enhancements in task preparation processes (Capa et al., 2013; Hall-McMaster et al., 2019). Although it is well established that performance-contingent rewards can lead to behavioral enhancements, evidence on the effects of potential losses on cognitive control is rather mixed and has not been examined during task-switching nor with developmental samples. Hence, whether potential rewards and losses affect cognitive control in a comparable manner is still an open issue.

### P3b as neural marker of incentive processing

Recent studies investigating the neural underpinnings of motivation by means of ERPs have proposed several components as putative markers of incentive processing (for a comprehensive overview, see Glazer et al., 2018). In particular, a P3b-like component, a centroparietal positivity that emerges between 300 and 500 ms after incentive presentation, often has been utilized as an indicator of incentive processing. Already in 1983, Begleiter and colleagues showed that the amplitude of the P3b component significantly differed among equiprobable, task-relevant visual stimuli depending on their incentive value and proposed that the P3b also may reflect a reaction to the incentive properties of stimuli (Begleiter et al., 1983). Similaly, Polich (2007, 2012) argued that the P3b reflects the amount of attentional resources allocated to the processing of motivationally salient information and the respective updating of working memory contents. In line with this, several ERP studies that examined motivational incentives in adult samples have reported increased P3b amplitudes after the presentation of incentives, with the P3b being larger for rewards relative to losses (Angus et al., 2017; Broyd et al., 2012; Flores et al., 2015; Zhang et al., 2017; Zheng et al., 2017) and neutral conditions (Giustiniani et al., 2020; Pornpattananangkul & Nusslock, 2015) and larger for higher relative to lower rewards (Goldstein et al., 2006).

For instance, in the study by Goldstein et al. (2006), the P3b amplitude varied symmetrically with reward magnitude. Likewise, Pfabigan et al. (2014) employed a monetary incentive delay (MID) task to assess incentive processing in a combined ERP and fMRI study and found higher P3b amplitudes for gain anticipation relative to loss and neutral trials. Interestingly, this valence-specific modulation of P3b during the anticipation phase was related to higher activation levels in the ventral striatum, which has been usually associated with incentive processing (see above). Additionally, there is evidence from studies in the domain of memory research showing that larger monetary incentives were associated with larger P3b amplitudes, suggesting an increased engagement of executive mechanisms during cue updating. Moreover, these larger P3b amplitudes were predictive of successful recollection from memory (Gruber and Otten, 2010; Halsband, Ferdinand, Bridger, & Mecklinger, 2012).

### The present study

Based on these theoretical and empirical foundations, the present study was designed to investigate how the prospect of performance-contingent incentives of different valence and magnitude may modulate incentive processing and cognitive control performance across early, middle, and late adolescence. Of special interest was whether and how the increased susceptibility to incentives during (middle) adolescence might be translated into a significant developmental advantage when linked to performance goals, facilitating the effective implementation of cognitive control. We employed an incentivized version of a cued task-switching paradigm combined with an ERP approach. In line with previous literature, we focused on the P3b after the presentation of high and low gain and loss cues as a neural indicator of incentive processing. Additionally, we examined the impact of these incentives on performance during task-switching, especially with respect to global and local switch costs. Our hypotheses were that potential gains, relative to losses, would lead to increased preparatory updating processes as reflected in the P3b response, also influenced by the respective magnitude of incentive expected. Because incentives have been found to improve task preparation in adult samples, we anticipated performance in the switching task to be modulated by the incentives also in our adolescent sample. Specifically, in line with previous literature suggesting mid-adolescence as a key developmental period in which reward drive is heightened, we expected that mid-adolescents should be especially sensitive to incentive cues, particularly high gains, resulting in respective performance benefits on the behavioral level. In summary, the primary goals of the present study were to examine (1) whether there are developmental differences between early-, mid-, and late-adolescents in the processing of the magnitude and the valence of incentives, and (2) whether these different types of incentives differentially influence the performance in simple cognitive tasks and switching between them.

## Method

### Participants

A total of 146 adolescents took part in this study and were divided into three age groups: early (10–12 years), mid (13–15 years), and late adolescents (16–18 years). All participants had normal or corrected-to-normal vision and were free of acute neurological or psychiatric disorders according to self-reports and reports of their legal guardians. The participants received 24 € for their participation. Informed consent was signed by participants or their legal guardians. Thirty-six participants had to be excluded from the analyses, because for one or more conditions there were less than 15 artefact-free trials for the ERP analyses (eleven 10–12-year-olds, twelve 13–15 year-olds, and thirteen 16–18-year-olds). Also, to secure active participation in the task, each participant’s response pattern in the single and mixed blocks of the task-switching task was checked against chance performance by means of a χ^2^ test (*α* = 0.05, *df* = 1). For this reason, nine additional participants (five 10–12-year-olds, three 13–15-year-olds, and one 16–18-year-old) had to be excluded. An overview over the effective sample (*n* = 104) can be found in Table 1. Note that, according to an a priori computation of sample size using G*Power, a total of 87 participants were required to reliably detect a medium effect size of 0.25, given an alpha level of 0.05, and a statistical power of 0.80 (Faul et al., 2007). Thus, despite the dropout of participants, our effective sample was large enough to detect the relevant effects.

**Table 1:** Sample characteristics

	10-12 year-olds	Age group 13-15 year-olds	16-18 year-olds
n (female/ male)	29 (11/18)	40 (18/22)	35 (21/14)
Mean age (years) (*SD*)	11.68 (.95)	14.46 (.88)	17.56 (.79)
DSST (ms) (*SD*)	1607 (252)	1371 (307)	1191 (217)
KFT (% correct items) (*SD*)	.39 (.14)	.55 (.17)	.69 (.20)
APM (% correct items) (*SD*)	.32 (.15)	.41 (.16)	.55 (.14)

*DSST* Digit Symbol Substitution Test (adapted from Wechsler, 2008), *APM* Raven Advanced Progressive Matrices Test (Raven et al., 1985), *KFT* Kognitiver Fähigkeits-Test für 4. bis 12. Klassen, Revision: KFT 4-12CR (Heller & Perleth, 2000); SD = standard deviation

To assess cognitive abilities, all participants performed computerized versions of a speeded Digit Symbol Substitution Test (DSST; adapted from Wechsler, 2008) as a marker of perceptual speed and the Raven Advanced Progressive Matrices Test (APM; Raven, Court, & Raven, 1985) as a marker of fluid intelligence. Additionally, participants completed an adapted version of the Word Puzzle from a German test for cognitive abilities for children and adolescents from 9–18 years (KFT 4-12CR, Heller & Perleth, 2000) as a marker of crystallized intelligence. As shown in Table 1, all tests showed the expected linear improvement with age (DSST: *F*(2,101) = 19.8, *p* < 0.01; APM: *F*(2,101) = 19.2, *p* < 0.01; KFT: *F*(2,99) = 22.9, *p* < 0.01).

### Task, stimuli, and procedure

Participants performed a cued task-switching paradigm with explicit cues indicating the upcoming task (task cues) as well as preceding cues signaling the incentive condition of the current trial (incentive cues). The task cue “FO” (for German “Form,” i.e., shape) indicated that subjects had to decide whether a stimulus depicted an inanimate object or an animal. The task cue “FA” (for German “Farbe,” i.e., color) signaled that participants should decide whether the stimulus was presented in greyscale or color. Subjects were instructed to respond as fast as possible to the target by pressing the respective button on a response box placed in front of the computer screen. All presented targets were bivalent, meaning they could both be judged with respect to their color as well as their shape. Also, overlapping response mappings were employed for the two tasks sets, resulting in high interference (Jamadar et al., 2015). As target stimuli, we used adapted stimuli from the databases of Rossion and Pourtois (2004) as well as Snodgrass and Vanderwart (1980).

**Fig. 1:**
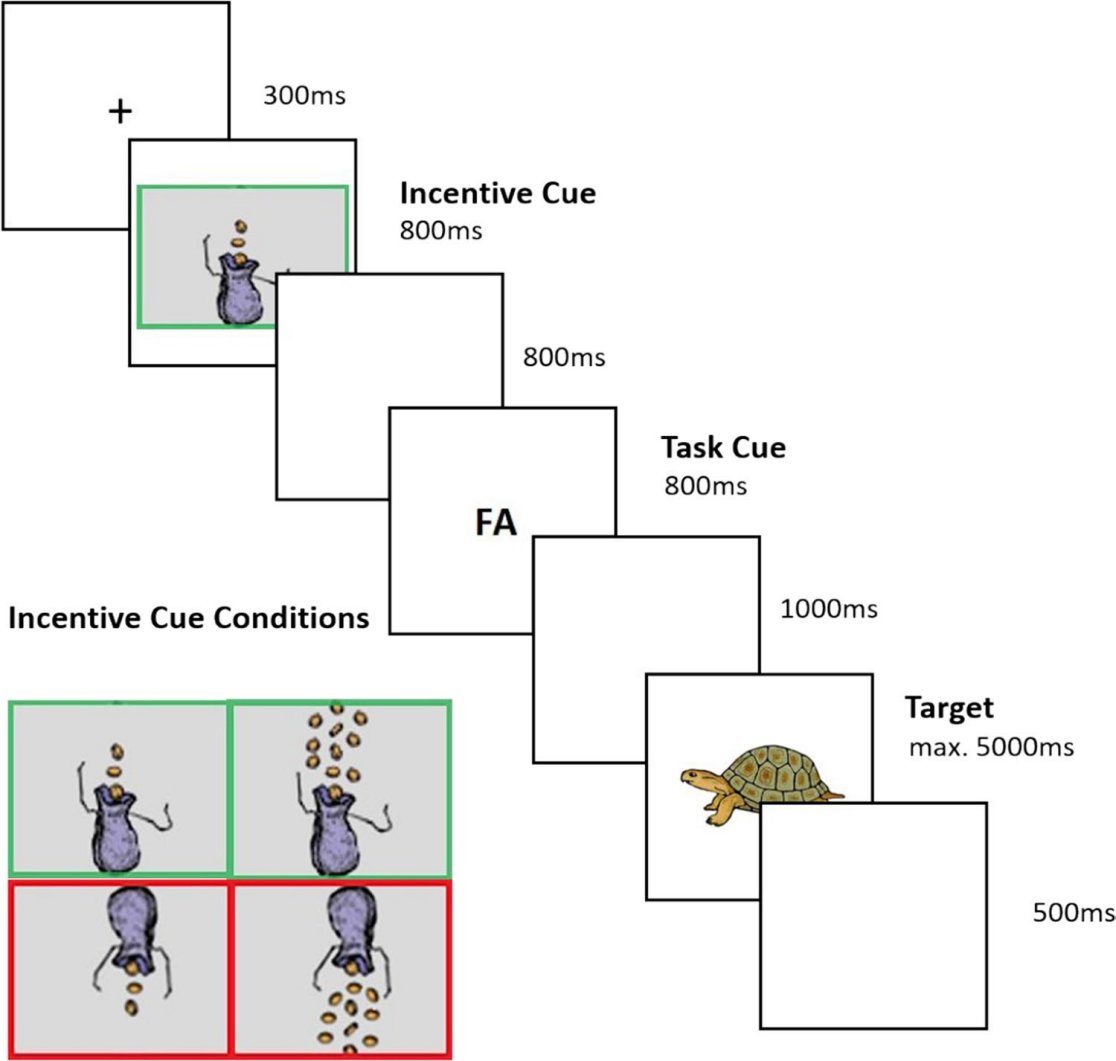
Trial procedure and incentive cue conditions in the task-switching task

Before the task cue, participants were presented with one of four different incentive cues. To examine effects of valence and magnitude of incentives separately, participants saw depictions of money bags that either lost or gained a small or large amount of gold coins. Thus, in total there were four motivational cues varying in incentive valence (i.e., gains vs. losses) and incentive magnitude (low vs. high; Fig. 1). For the images of the money bags, we used adapted versions from the study by Schmitt et al. (2015).

Participants were instructed that incentive cues signaled a bonus that they could achieve or lose in the current trial, depending on their performance in the pending task. That is, by answering both fast and accurately they could gain a small or large amount of coins or avoid losing a small or large amount of coins, respectively. We employed this combined performance criterion to prevent participants from strategical speed-accuracy trade-offs, i.e., intentionally slowing down their response for increasing accuracy. After completing the experiment, the final bonus could be exchanged for a reward, choosing from a variety of sweets and gift items, which differed in attractiveness depending on the achieved performance (e.g., school supplies, brain teasers, fidget toys, accessories, bonbons, etc.). Note that participants were presented with the available rewards only after the completion of the task to control for age differences of incentive preferences and the perceived value of the available rewards.

In the experimental phase, participants performed a total of 18 blocks, each containing 32 trials. Six of those blocks were single-task blocks, in which participants only performed trials of the same task (i.e., 3 blocks of the color and the shape task), and 12 were mixed-task blocks, in which subjects had to perform trials of both the shape as well as the color task. In the mixed-task blocks, there was an equal number of repetition trials, where subjects performed the same task as in the preceding trial, and switch trials, in which participants performed a different task than in the preceding trial. The presentation of all blocks and response configurations was counterbalanced across subjects. After the completion of each block, participants received feedback on their performance, reporting both accuracy and mean response speed. Importantly, to focus exclusively on the anticipatory effects of performance-contingent incentives, no feedback information about the amount of the achieved bonus was given throughout the task. Before the experimental phase, participants completed an additional set of three practice blocks, one single-task block of each task condition and a mixed-task block.

Each trial started with the presentation of a fixation cross for 300 ms, followed by the incentive cue (800 ms), a blank screen (800 ms), the task cue (800 ms), a second blank screen (1,000 ms), and the target stimulus. The target stimulus was presented until the participants’ response or for a maximum of 5,000 ms. After the target stimulus, there was another blank screen for 500 ms, followed by the start of the next trial. For an overview of the task procedure, see Fig. 1.

### Data recording and pre-processing

The task was performed on a Dell Optiplex 9010 computer in an electrically shielded chamber. Responses were registered with an external response pad (Response Pad RB-844, Cedrus Corporation). For the behavioral data, trials with timeouts or reaction times faster than 100 ms were excluded from the individual data sets.

During the task, the EEG was recorded from 59 Ag/AgCl active electrodes embedded in an elastic cap (actiCAP, Brain Products, Germany) and recording locations were based on the extended 10–20 System (Jasper, 1958). EEG was amplified from DC to 100 Hz at a sampling rate of 500 Hz using Brain Vision Recorder (Brain Products, Germany). An additional electrode on the left mastoid served as reference, the ground electrode was placed at AFz. To control for eye movements, the electrooculogram (EOG) was recorded from four electrodes at the outer ocular canthi and right suborbital and supraorbital ridges. Impedances were kept below 20 kΩ. Offline processing of EEG data was conducted using Brain Vision Analyzer (Brain Products, Germany). Data were band-pass filtered from 0.1-30 Hz offline and re-referenced to linked mastoids. Eye movements were corrected by an independent component analysis approach. If bad channels were channels of interest (i.e., midline electrodes Fz, Cz, or Pz), the respective participant was excluded from data analyses. Trials were averaged using EEProbe (ANT Neuro). Here, trials including artifacts were excluded from averaging if the standard deviation in a 200-ms time interval was larger than 20 μV. A 100-ms prestimulus baseline was used for all ERP averages. A minimum of 15 artefact-free trials per condition was implemented as a criterion for inclusion in the ERP analyses. Following the artefact rejection 48 trials (*SD* = 18) were included on average for each condition type per participant.

The analysis of EEG data was based on ERPs time-locked to the presentation of the motivational cues, including a 100-ms prestimulus baseline. ERP epochs ranged from 200 ms before to 1,100 ms after the presentation of the motivational cue. We examined the P3b in the motivational cue interval by means of mean amplitudes. It was measured between 250 ms and 400 ms for all age groups. This time window was selected according to our previous study with adults (Schmitt et al., 2015) and based on visual inspection of the waveforms. The P3b is commonly examined at Pz where it is usually found to be largest (Polich, 2004, 2007). However, sometimes frontal shifts of this component are found and thought to mirror effort in terms of an additional recruitment of frontal networks (Ferdinand et al. 2016). For this reason, we analyzed P3b at three midline electrodes ranging from anterior to posterior, i.e., Fz, Cz, and Pz.

### Statistical analysis

Behavioral and ERP data were analyzed using repeated measures analyses of variance (ANOVAs) with an alpha level of 0.05. For all analyses, the between-subjects factor Age Group (10–12-year-olds, 13–15-year-olds, 16–18-years-olds) and the within-subjects factors Incentive Valence (gain, loss) and Incentive Magnitude (high, low) were used. Additionally, for the behavioral analyses, the factor Trial Type (single, repeat, switch) was reflected in two *a priori* orthogonal contrasts, the first contrast compared the mean performance in mixed trials versus single trials (reflecting global switch costs), and the second contrast compared the mean performance in repeat versus switch trials (reflecting local switch costs). Behavioral analyses were performed on reaction times based only on accurate responses to ensure validity. Also, practice blocks and trials with timeout or RTs below 100 ms were excluded from the data analyses. To be able to compare data from the different age groups, reaction times were log-transformed to control for the different age groups’ reaction time levels (Kray, 2000; Meiran, 1996). To check for speed-accuracy trade-offs, we also performed the same analysis on mean accuracies.

For the analyses of ERP data, the factor Block (single, mixed) was used instead of Trial Type and the factor Anterior/Posterior (Fz, Cz, Pz) was included in addition to the other three factors, Age Group, Incentive Valence, and Incentive Magnitude, that entered the behavioral analysis. Greenhouse-Geisser correction for nonsphericity was applied whenever appropriate and epsilon-corrected *p*-values are reported together with uncorrected degrees of freedom. The Bonferroni correction was used for post-hoc testing.

## Results

### Behavioral results

**Fig. 2:**
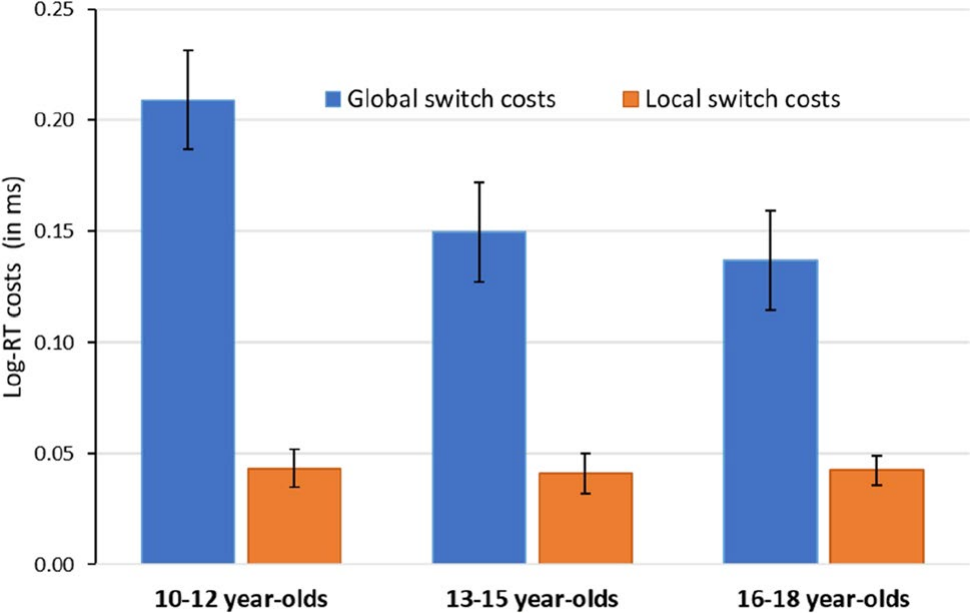
Global and local switch costs calculated on log-transformed reaction times for the three age groups

**Fig. 3:**
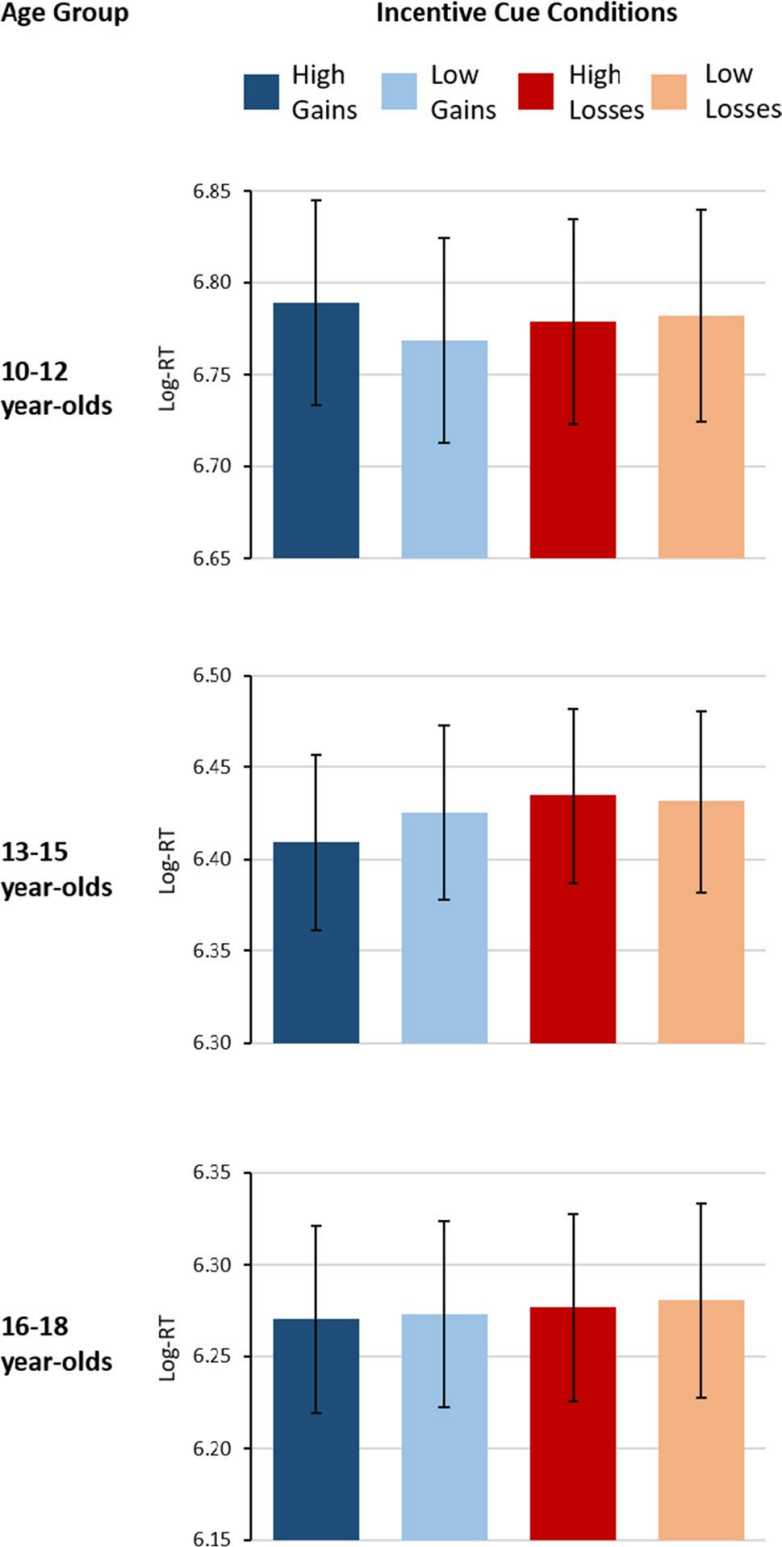
Log-transformed reaction times for high and low gains and losses for the three age groups

Mean reaction times and accuracies for all experimental conditions separately for the three age groups are presented in Table 2. Global switch and local switch costs, based on log-transformed data, are displayed in Fig. 2. Log-transformed reaction times for high and low gains and losses are displayed separately for the three age groups in Fig. 3.

**Table 2:** RTs and ACC in single, repeat, and switch trials as a function of incentive cue condition (high and low gains, high and low losses) separately for the three age groups

Age group	Incentive cue condition	RT						ACC					
		Single trials		Repeat trials RT		Switch trials RT		Single trials ACC		Repeat trials ACC		Switch trials ACC	
		*M*	*SD*	*M*	*SD*	*M*	*SD*	*M*	*SD*	*M*	*SD*	*M*	*SD*
10-12 year-olds	High gain	833	338	978	371	1029	385	.93	.06	.91	.06	.89	.08
	Low gain	809	324	982	372	988	353	.94	.06	.91	.07	.88	.08
	High loss	796	287	976	362	1036	394	.95	.06	.92	.06	.89	.08
	Low Loss	810	314	984	376	1032	376	.93	.05	.92	.05	.90	.07
13-15 year-olds	High gain	570	175	661	223	687	237	.96	.05	.94	.07	.93	.06
	Low gain	579	175	673	239	703	244	.97	.05	.98	.06	.92	.08
	High loss	584	172	679	234	709	259	.95	.05	.95	.06	.92	.06
	Low Loss	591	201	677	275	711	251	.96	.07	.94	.07	.92	.07
16-18 year-olds	High gain	495	106	567	200	594	223	.98	.03	.95	.04	.94	.05
	Low gain	495	107	571	193	592	217	.96	.03	.95	.04	.93	.04
	High loss	491	102	572	213	607	224	.97	.03	.96	.04	.94	.04
	Low Loss	499	102	574	204	606	253	.97	.03	.97	.03	.93	.05

*RT* reaction time in ms, *ACC* accuracy in percentage correct responses, *M* mean, *SD* standard deviation

ANOVA with the between-subjects factor Age Group (10–12 years, 13–15 years, 16–18 years) and the within-subjects factors Incentive Valence (gain, loss), Incentive Magnitude (high, low), and Trial Type (single, repeat, switch) was conducted on log-transformed reaction times. The ANOVA yielded main effects of Age Group (*F*(2,101) = 23.06, *p* < 0.001, *η*_*p*_^*2*^ = 0.31), indicating that 10–12-year-olds had overall longer reaction times than 13–15-year-olds (*M* = 0.35; *SD* = 0.07, *p* < 0.001) and longer reaction times than 16–18-year-olds (*M* = 0.51; *SD* = 0.08, *p* < 0.001), whereas the 13–15- and 16–18-year-olds did not differ (*p* = 0.101). Additionally, main effects of Incentive Valence (*F*(1,101) = 4.41, *p* = 0.038, *η*_*p*_^*2*^ = 0.04), Global switch costs (*F*(1,101) = 189.67, *p* < 0.001, *η*_*p*_^*2*^ = 0.65), and Local switch costs (*F*(1,101) = 76.58, *p* < 0.001, *η*_*p*_^*2*^ = 0.43), as well as an interaction between Age Group and Global switch costs (*F*(2,101) = 3.16, *p* = 0.047, *η*_*p*_^*2*^ = 0.06), suggesting that global switch costs decreased with increasing age, but no interaction between Age Group and local switch costs (*p* = 0.978) (Fig. 2)*.*

We also found a marginally significant interaction between Age Group, Incentive Valence, and Incentive Magnitude (*F*(2,101) = 2.92, *p* = 0.058, *η*_*p*_^*2*^ = 0.06). To dissolve this interaction, separate ANOVAs were conducted for each age group. Only the analyses of the 13-15 year-olds showed a main effect of Incentive Valence (*F*(1,39) = 7.97, *p* = 0.007, *η*_*p*_^*2*^ = 0.17), that was further modulated by a marginally significant interaction with Incentive Magnitude (*F*(1,39) = 3.35, *p* = 0.075, *η*_*p*_^*2*^ = 0.08). Post-hoc tests indicated that only the comparison between high gains and high losses was significant (*F*(1,39) = 11.00, *p* = 0.002, *η*_*p*_^*2*^ = 0.22), indicating faster responding in high gain than in high loss trials (Fig. 3).

To check for incentive-specific speed-accuracy trade-offs, we also performed an ANOVA on mean accuracies with the between-subjects factor Age Group (10–12 years, 13–15 years, 16–18 years) and the within-subjects factors Incentive Valence (gain, loss), Incentive Magnitude (high, low), and Trial Type (single, repeat, switch). This analysis yielded a main effect of Age Group (*F*(2,101) = 6.42, *p* = 0.002, *η*_*p*_^*2*^ = 0.11), indicating that the 10–12-year-olds had overall worse accuracy than the 13–15-year-olds (*M* = 0.03; *SD* = 0.01, *p* = 0.039) and the 16–18-year-olds (*M* = 0.04; *SD* = 0.01, *p* = 0.002), and a main effect of Trial Type (*F*(2,202) = 87.32, *p* < 0.001, *η*_*p*_^*2*^ = 0.46), reflecting significant global switch costs (*F*(1,101) = 97.91, *p* < 0.001, *η*_*p*_^*2*^ = 0.49) and local switch costs (*F*(1,101) = 73.02, *p* < 0.001, *η*_*p*_^*2*^ = 0.42). Additionally, the analysis yielded a marginally significant interaction between Incentive Valence and Trial Type (*F*(2,202) = 2.69, *p* = 0.070, *η*_*p*_^*2*^ = 0.03). However, when dissolving this interaction, no significant differences between the gain and loss condition emerged (all *p*-values >0.18). Importantly, no significant effects of Incentive Valence or Incentive Magnitude on accuracy were indicated (nor interactions between both and Age Group), suggesting no incentive-driven response biases related to accuracy.

### P3b in the motivational cue interval

**Fig. 4:**
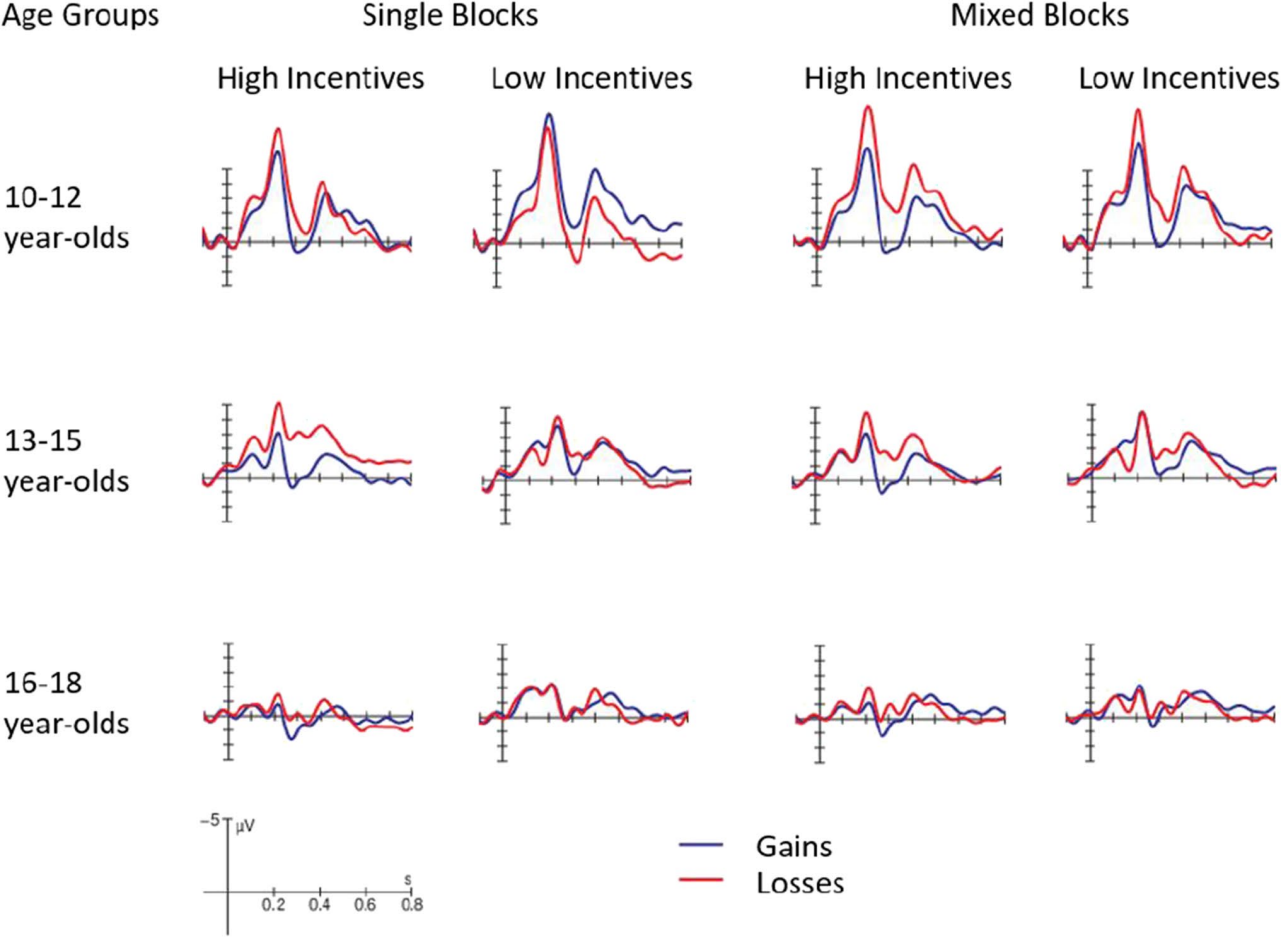
ERPs in the time interval after the incentive cue (gains vs. losses) at the Pz electrode for the three age groups, separately for single vs. mixed blocks and high vs. low incentives. (A low-pass filter of 12 Hz was applied for visualization only)

**Fig. 5:**
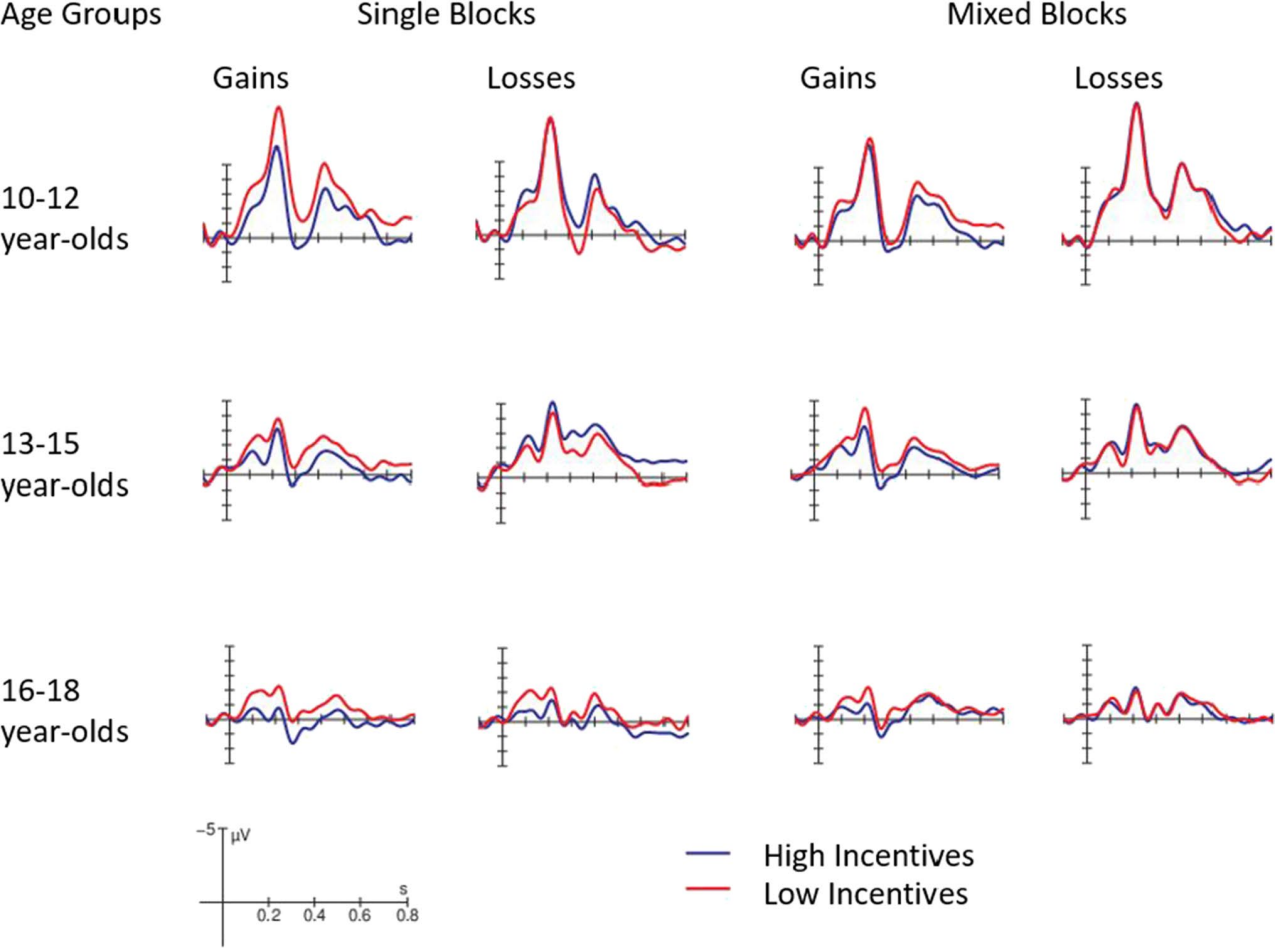
ERPs in the time interval after the incentive cue (high vs. low incentives) at the Pz electrode for the three age groups, separately for single vs. mixed blocks and gains vs. losses. (A low-pass filter of 12 Hz was applied for visualization only)

Figures 4 and 5 display ERPs in the time interval after the motivational cue at electrode Pz for all three age groups separately for single versus mixed blocks. For a better visualization of the found effects, we displayed the ERP data in two ways, so that Fig. 4 allows an easy comparison between gains and losses and Fig. 5 between high and low incentives.

The ANOVA with the between-subjects factor Age Group (10–12 years, 13–15 years, 16–18 years) and the within-subjects factors Incentive Valence (gain, loss), Incentive Magnitude (high, low), Block Type (single, mixed), and Anterior/Posterior (Fz, Cz, Pz) was conducted on P3b mean amplitudes. The results indicated main effects of Incentive Valence (*F*(1,101) = 6.556, *p* = 0.012, *η*_*p*_^*2*^ = 0.06) and Anterior/Posterior (*F*(2,202) = 45.09, *p* < 0.001, *η*_*p*_^*2*^ = 0.31), as well as two-way interactions between Incentive Valence and Incentive Magnitude (*F*(1,101) = 12.22, *p* = 0.001, *η*_*p*_^*2*^ = 0.11), Incentive Valence and Block Type (*F*(1,101) = 5.12, *p* = 0.026, *η*_*p*_^*2*^ = 0.05), and Incentive Valence and Anterior/Posterior (*F*(2,202) = 29.98, *p* < 0.001, *η*_*p*_^*2*^ = 0.23). Moreover, we found a number of three-way interactions between Age Group, Incentive Valence, and Block Type (*F*(2,101) = 5.89, *p* = 0.004, *η*_*p*_^*2*^ = 0.10), Age Group, Incentive Valence, and Anterior/Posterior (*F*(4,202) = 4.15, *p* = 0.003, *η*_*p*_^*2*^ = 0.08), Incentive Valence, Incentive Magnitude, and Block Type (*F*(1,101) = 5.34, *p* = 0.023, *η*_*p*_^*2*^ = 0.05), and Incentive Valence, Incentive Magnitude, and Anterior/Posterior (*F*(2,202) = 6.20, *p* = 0.002, *η*_*p*_^*2*^ = 0.06).

To better understand the nature of these interactions, separate ANOVAs were conducted for each age group and block type. Furthermore, because the topography of the P3b was largest at electrode Pz for all age groups and conditions, we focused these ANOVAs on Pz (Figs. 4 and 5).

#### Single blocks

The ANOVA for the 10–12-year-olds showed an interaction between Incentive Valence and Incentive Magnitude (*F*(1,28) = 6.91, *p* = 0.014, *η*_*p*_^*2*^ = 0.20). Post-hoc analyses showed that the comparisons between high and low gains (*F*(1,28) = 5.74, *p* = 0.096, *η*_*p*_^*2*^ = 0.17; Fig. 5, upper row) and between low gains and losses (*F*(1,28) = 5.72, *p* = 0.096, *η*_*p*_^*2*^ = 0.17; Fig. 4, upper row) were marginally not significant (all other comparisons: *p* > 0.72). For the 13–15-year-olds, the ANOVA yielded a main effect for Incentive Valence (*F*(1,39) = 16.33, *p* < 0.001, *η*_*p*_^*2*^ = 0.30), that was also qualified by an interaction with Incentive Magnitude (*F*(1,39) = 9.32, *p* = 0.004, *η*_*p*_^*2*^ = 0.19). This was explained by a larger P3b amplitude for high gains than high losses (*F*(1,39) = 30.64, *p* < 0.001, *η*_*p*_^*2*^ = 0.44), whereas this was not the case for low incentives (*p* > 0.90). Additionally, in tendency the P3b amplitude was larger for high than low gains (*F*(1,39) = 6.01, *p* = 0.076, *η*_*p*_^*2*^ = 0.13; Fig. 4, middle row). For the 16–18-year-olds, in contrast, the analysis only showed a main effect for Incentive Magnitude (*F*(1,34) = 8.45, *p* = 0.006, *η*_*p*_^*2*^ = 0.20), suggesting a larger P3b amplitude for high than low incentives (Fig. 5, lower row).

#### Mixed blocks

All age groups showed a main effect of Incentive Valence with a larger P3b amplitude for gains than losses (10–12 years: *F*(1,28) = 24.14, *p* < 0.001, *η*_*p*_^*2*^ = 0.46; 13–15 years: *F*(1,39) = 16.43, *p* < 0.001, *η*_*p*_^*2*^ = 0.30; 16-18 years: *F*(1,34) = 4.85, *p* = 0.034, *η*_*p*_^*2*^ = 0.13; Fig. 4). Only for 13–15-year-olds, this effect was further qualified by Incentive Magnitude (*F*(1,39) = 4.19, *p* = 0.047, *η*_*p*_^*2*^ = 0.10), indicating that the difference between gains and losses was more pronounced for high incentives (*F*(1,39) = 16.67, *p* < 0.001, *η*_*p*_^*2*^ = 0.30) than low incentives (*F*(1,39) = 6.58, *p* = 0.056, *η*_*p*_^*2*^ = 0.14), and that the P3b was larger for high compared with low gains (*F*(1,39) = 5.58, *p* = 0.092, *η*_*p*_^*2*^ = 0.13; Fig. 4, middle row).

## Discussion

The purpose of the present study was to examine age differences in incentive sensitivity throughout adolescent development, in particular in the neural processing of the valence and magnitude of incentive cues and the corresponding task performance and switching behavior. We compared early-, mid-, and late-adolescents by means of ERPs to assess motivational cue processing of high and low potential gains and losses and examined whether the valence of incentives, the magnitude, or both also corresponded to performance differences in task-switching costs. Given previous evidence for higher reward sensitivity in mid-adolescence (Casey et al., 2008; Shulman et al., 2016), we expected that primarily mid-adolescents compared with early- or late-adolescents would be especially sensitive to high gains as reflected in a larger P3b response. A further question was whether differences in incentive processing would impact behavior in a task-switching task. Our findings suggest differential effects at the neurophysiological and behavioral level. The analysis of the P3b showed age-related differences in the processing of incentive cues that also varied with cognitive control demands, measured as the difference between single and mixed task blocks. In contrast, the incentive manipulation had no impact on age-related differences in the efficiency of cognitive control implementation in terms of task switching costs, but rather a general effect on response speed.

### Developmental differences in the processing of incentive cues

To examine how incentives were processed, we focused our analyses on the P3b in the time interval after the presentation of the incentive cues (before the task cues were presented). The P3b component has been considered to be an indicator of the amount of attentional resources allocated to the processing of motivationally salient information and the respective updating of working memory (Polich, 2007, 2012). Overall, all age groups displayed a parietally distributed P3b indicating that they all allocated resources to process the properties of the incentive cues. Moreover, at electrode Pz where the effects were maximal for all age groups, we found age-related differences in the processing of valence and magnitude of the incentives that also differed across task demands (induced by performing only one task in a block or by the need to switch between two tasks throughout a block).

The most prominent finding of this study was that mid-adolescents, compared with early- and late-adolescents, were indeed particularly sensitive to both features of incentive cues, i.e., their valence and magnitude. More specifically, mid-adolescents showed a larger P3b for gains than losses, and this effect was more pronounced for high than low incentives. Interestingly, this pattern of findings was independent of cognitive control demands in this age group, as it was found in single as well as in mixed task blocks. The higher sensitivity to motivational cues in middle adolescence has been interpreted as a fast maturation of the socio-emotional system in recent dual-system models (Shulman et al., 2016). It also is in line with empirical evidence (Galvan et al., 2006), suggesting that mid-adolescents compared with children and young adults show the largest differences in neuronal recruitment of the nucleus accumbens, a region linked to reward processing, between small, medium, and large monetary gains. Although the pattern of brain activation in this study (Galvan et al., 2006) was similar for adults and mid-adolescents, the differences in the neuronal recruitment in the three reward conditions was less pronounced and indifferent in children. Similarly, in our present study, the sensitivity to incentives as reflected in the updating of cue information was less differentiated in late-adolescents. However, in the study by Galvan et al. (2006) only neuronal changes to gains and not to losses were measured and the processing of cues was independent of task performance.

In contrast to mid-adolescents, the demands on cognitive control appeared to influence the processing of incentive cues in early- and late-adolescents. In single tasks that require less cognitive control, we found that early-adolescents displayed a tendency for a larger P3b in trials with high gain and low loss incentives. Hence, whereas mid-adolescents focused on processing the magnitude of incentives clearly prioritizing high gains over high losses, early adolescents yielded a less clear pattern of preference. Interestingly, in the more demanding switching situation, the age differences that were found in the single blocks almost vanished. Here, all age groups had larger P3b amplitudes after gain than loss incentives reflecting more updating processes after gain cues. On top of that, mid-adolescents still yielded a preference of high gains over low gains that was not evident in the other age groups. Thus, early- as well as late-adolescents shifted their focus toward updating after gain cues in the more demanding condition, as reflected in larger P3b amplitude for gains than losses. Hence, one could speculate that if cognitive control demands are increased, individuals might prefer a simpler pattern of incentive processing, namely favoring gains over losses. This line of reasoning is in accordance with studies reporting a relation between a low working memory capacity and a decreased use of controlled processing strategies together with an increased use of easier automatic processing (Hofmann et al., 2008; Kane et al., 2001). These findings also hint at the possibility, that the enhanced sensitivity to high gains in mid-adolescents might be due to a strategic as well as a more automatic, biologically explained, bias: In addition to a possibly strategic shift of resource allocation towards the processing of high gains when control demands are low, a more basic bias like a general hyperactivity of the reward system might be at work when control demands are high.

Notably, the stimuli selected as cues for the incentive conditions shared a great perceptual similarity, allowing for isolating ERP effects related to the actual incentive processing from effects linked to differences in lower-level salience processing. Hence, the obtained modulations cannot be attributed to perceptual changes, but rather reflect underlying motivational and attentional processes. Indeed, a number of previous studies have linked incentive-induced increases in P3b amplitudes to an increased allocation of attentional resources in the prospect of incentives (e.g., Gruber & Otten, 2010; Halsband et al., 2012; Polich, 2007, 2012).

Taken together, mid-adolescents displayed a clear sensitivity to motivational cues signaling the prospect of high gains in single as well as in mixed blocks, in line with our hypothesis. However, we did not find a general preference for gains over losses and for high over low incentives in all age groups. Instead, late-adolescents showed a preference for high over low incentives in single blocks, irrespective of whether they were potential gains or losses, while early-adolescents yielded a rather inconsistent pattern of results. In mixed blocks, which are much more demanding on cognitive control than single blocks, all age groups seemed to revert to a simpler pattern and preferably processed gains over losses.

### Performance in the task-switching task

In the behavioral data, we found that the 10–12-year-old age group had overall longer reaction times than the 13–15 and 16–18 age groups, whereas the 13–15 and 16–18 age groups did not differ. This finding indicates the expected increase in general processing speed throughout adolescence. Consistent with the literature, we also found significant global and local switch costs in all age groups. While global switch costs, usually interpreted as reflecting the cost of maintaining multiple task-sets and having to select between them, decreased with increasing age, local switch costs, i.e., the costs for switching itself, appeared to be age-invariant (Crone et al., 2006; Karbach & Kray, 2007; Kray et al., 2004; Manzi et al., 2011; Reimers & Maylor, 2005). This pattern of results is usually construed as an improving ability to update and maintain task-set representations in working memory and to select between them, while the ability to switch is considered as relatively stable. Importantly, these age effects were obtained after statistically controlling for differences in processing speed between the three age groups (Kray & Lindenberger, 2000).

In addition to replicating age-related findings in global and local switch costs, of most interest in our study was the putative effects of incentives on task performance and cognitive control. Here, we found that only mid-adolescents’ reaction times were sensitive to the valence of the incentives. They responded faster in trials where potential gains could be obtained than in trials with potential losses. This effect was tendentially increased when the incentive magnitude was high. This finding is in line with the idea of an increased reward sensitivity in mid-adolescence and also matches our ERP results in this age group. Hence, taking our ERP findings into account, this observed facilitation of task performance could be associated with the heightened P3b response after high gains also found in this age group. Hence, our findings corroborate the assumptions on a heightened reward sensitivity during mid-adolescence and support previous findings, suggesting that this sensitivity may actually result in a developmental advantage over other age groups, especially when the attainment of incentives is bound to task performance (Strang & Pollak, 2014; van Duijvenvoorde et al., 2016). Importantly, this observed facilitation of performance did not appear to be a result of strategical speed-accuracy trade-offs.

However, this age-specific behavioral advantage in the face of high gains was only found on a general performance level by speeding up all responses in trials with potential high gains, irrespective of the actual cognitive control demands. Hence, in contrast to our expectations, we found no specific interaction between the motivational incentives and our measures of cognitive control, i.e., global and local switch costs. Instead, the incentive-induced performance improvements in mid-adolescents appeared to rather be the result of a global increase in vigor and motivational drive than being bound to task-switching performance per se. This was surprising because there are studies employing incentivized TS paradigms in adult samples that have reported selective decreases in task-switching costs in anticipation of incentives (Kleinsorge & Rinkenauer, 2012; Otto & Vassena, 2020; Shen & Chun, 2011; Umemoto and Holroyd, 2015, b). However, Kleinsorge and Rinkenauer (2012) found a reduction of global switch costs only when the assignment of incentives to the respective tasks was fixed, whereas implementing a variable association between incentives and tasks merely resulted in a rather global facilitation of task performance, as in our study. In a similar vein, Shen and Chun (2011) found incentive-related reductions in switching costs only in trials in which the incentives increased relative to the previous trial. Moreover, other studies employing incentivized TS paradigms in adult samples did not yield any selective modulations of task-switching costs by incentives. For example, both Capa et al. (2013) and Cubillo et al. (2019) reported solely a global performance facilitation by incentives, in terms of heightened reaction speed, in line with the current findings. To the authors’ best knowledge, studies directly examining the effects of different amounts of gain and loss incentives on task-switching ability in the course of adolescence are fully missing. Hence, no direct comparisons with other developmental samples can be made.

### Limitations and outlook

Although incentives successfully enhanced overall task performance in mid-adolescents, no selective interaction between incentives and cognitive control, in terms of global or local switch costs, was found. Taking into account the findings of Soutschek et al. (2014), suggesting ceiling effects in behavioral improvement when combining both incentive and task-informative cueing, it is possible that, given the relatively prolonged cue-target-interval (CTI) applied in the current design, participants had enough time to effectively process the task cues before target presentation, taking performance to a level where no further decrease in task-switching costs was possible. Thus, even if incentive cues did indeed foster the preparatory utilization of subsequent task-cue information, as reported in previous studies (Chiew & Braver, 2016), this interaction might not have become evident in task-switching costs, because there was no room for further improvement. Please note that accuracies were generally quite high in all age groups (Table 2). Future studies could address this possibility by applying a shorter CTI or using an adaptive performance criterion to ensure high effort levels across participants.

Moreover, it is noteworthy that, due to later dropouts in the current study, the early adolescent group turned out to be predominately male, while the late adolescent group was predominately female (see effective sample in Table 1). Given the substantial sex differences in pubertal development (Mendle, Beltz, Carter, & Dorn, 2019), the question may arise as to whether this unequal sex distribution has contributed to the observed age effects. Indeed, little is known so far about the impact of sex on the brain’s responsiveness to incentives during adolescence (for recent findings, see Greimel et al., 2018; Wang, Liu, & Shi, 2017). Interestingly, however, in the age group of mid adolescents, who appeared to be most sensitive toward the incentive manipulation in the current study, male and female participants were approximately equally represented. Hence, the heightened reward sensitivity particularly observed within this age group cannot be attributed to confounding sex differences.

## Conclusions

This study examined the processing of high and low gain and loss incentives, as well as their impact on performance in a task-switching task in early-, mid-, and late adolescents. Our results corroborate the idea of a special sensitivity to high rewards during mid-adolescence as proposed by several theories on adolescent development (Casey et al., 2008; Steinberg, 2008). Moreover, they demonstrated that this sensitivity not only implicates a potential risk for adolescent development by contributing to risky behavior but also can lead to benefits in task performance.
